# Association of FGF Receptor-2rs2981578 SNP with breast cancer in women at the University of Gondar Comprehensive Specialized Hospital, Ethiopia

**DOI:** 10.1371/journal.pone.0327034

**Published:** 2025-08-01

**Authors:** Mihiret Bogale, Tiget Ayelgn Mengstie, Ephrem Tadesse, Tadesse Asmamaw Dejenie, Banchamlak Teferi, Melkamu Siferih, Mulualem Nibret Takle, Tseganesh Asefa, Gashaw Dessie, Winta Tesfaye, Endeshaw Asaye Kindie, Hiwot Tezera Endale, Kibur Hunie Tesfa, Birhanu Ayelign, Tewodros Shibabaw

**Affiliations:** 1 Department of Biomedical Sciences, School of Medicine, College of Medicine and Health Sciences, Wollo University, Dessie, Ethiopia; 2 Department of Biochemistry, School of Medicine, College of Medicine and Health Sciences, University of Gondar, Gondar, Ethiopia; 3 Internal Medicine, School of Medicine, College of Medicine and Health Sciences, University of Gondar, Gondar, Ethiopia; 4 Department of Clinical Pharmacy, School of Pharmacy, College of Medicine and Health Sciences, University of Gondar, Gondar, Ethiopia; 5 Department of Obstetrics and Gynecology, School of Medicine, Debre Markos University, Debre Markos, Ethiopia; 6 Department of Medical Nursing, School of Nursing, College of Medicine and Health Sciences, University of Gondar, Gondar, Ethiopia; 7 Department of Human Physiology, School of Medicine, College of Medicine and Health Sciences, University of Gondar, Gondar, Ethiopia; 8 Department of Human Pathology, School of Medicine, College of Medicine and Health Sciences, University of Gondar, Gondar, Ethiopia; 9 Department of Immunology and Molecular Biology, School of Biomedical and Laboratory Science, College of Medicine and Health Science, University of Gondar, Gondar, Ethiopia; Nigerian Institute of Medical Research, NIGERIA

## Abstract

**Introduction:**

Fibroblast Growth Factor Receptor-2 single nucleotide polymorphisms are implicated in breast cancer development. However, there are inconsistencies and contradictions in the reports. The current study aimed to determine the association between Fibroblast Growth Factor Receptor-2 rs2981578 single nucleotide polymorphism and breast cancer among female breast cancer patients.

**Methods:**

A case-control study was conducted between August 2023 and April 2024 at University of Gondar Comprehensive Specialized Hospital, Gondar, and Northwest Ethiopia. Semi-structured was adapted to collect socio-demographic character and anthropometric measurement and 5 ml of blood were drawn from each randomly selected participant for molecular analysis. The data was entered into Epi-Data-7.2 and exported to SPSS-25. Binary logistic regression model was used to look into the association between various factors. Statistical significance was regarded as a p-value of < 0.05.

**Results:**

The Fibroblast Growth Factor Receptor-2 Single Nucleotide Polymorphisms rs2981578 AA genotype and A allele were more frequent in breast cancer patients than in controls (37% vs. 17% and 58% vs 42%; the chi-square p = 0.025 and p = 0.009, respectively). Individuals with the AA genotype and A allele were more prone to breast cancer (OR = 3.3; 95% CI: 1.293–8.538, and OR =1.88; 95% CI: 1.173, 3.029, respectively) than those with the wild type GG genotype. The risk allele correlated with the late TNM stage (COR = 3.41, 95% CI: 1.07–10.88).

**Conclusion:**

The AA genotype and the A allele of the Fibroblast Growth Factor Receptor-2 Single Nucleotide Polymorphisms rs2981578 gene are more prevalent among women with breast cancer. They are correlated with increased susceptibility to breast cancer and a worsening of its stage.

## Introduction

According to the Global Burden of Cancer Study (GLOBOCAN) 2020 report, breast cancer remains the most frequent cancer among women, with 2.3 million (24.5%) cases and 684,996 (15.5%) deaths worldwide. According to the same report, it is the fifth biggest cause of morbidity worldwide. North America, Europe, and Oceania have the highest, whereas Africa and Asia have the lowest incidence rates. However, low and middle-income nations reported an increase in the incidence [1]. It is anticipated that by 2040, there will be over 3 million new cases of breast cancer showing an increase of more than 40% and a 50% increase to 1 million in breast cancer-related deaths [2].Across Africa, there were 186,598 newly reported cases and 85,787 fatalities in 2020, and projected to double by 2040 [3]. Ethiopia’s breast cancer incidence was 32,970 new cases, with a fatality rate of around 16,133 (31.9%) among female cancers [1].

Despite the exact mechanism behind remains unclear, breast cancer is a multifactorial disease, with various sociodemographic, behavioral, genetic, environmental exposures, and reproductive risk factors all contributing to susceptibility [4].Overall, hereditary factors have been identified as the most important characteristics in genetic variants of vulnerable genes in breast cancer patients. To date, various genome-wide association studies have revealed genetic variations that increase the risk of developing breast cancer. Such risk variant mutations possess varying penetrance and produce various malignant phenotypes. High-risk genes and single-nucleotide polymorphisms (SNPs) are associated with up to more than 50% lifetime risk and progression of breast cancer and have a large individual effect, although they are relatively uncommon in the general population [5]. Moderate penetrance genes have 20–50% lifetime breast cancer risk [6,7].In addition, 80 common low penetrance genes including, FGFR2 were identified by genome-wide association studies (GWAS), and smaller studies were also explained as having a 20% lifetime risk of breast cancer [8,9].

The Fibroblast Growth Factor Receptor-2 (FGFR2) gene is located on the longer arm of chromosome 10(10q26.13) and encompasses 120 kb consisting of 26 exons and 38 introns. The gene encodes Fibroblast Growth Factor Receptor 2, which acts as receptor tyrosine kinase tasked with relaying signals from fibroblast growth factors (FGFs). The Association involving the fibroblast growth factor and its corresponding receptor, Fibroblast Growth Factor Receptor-2regulates enormous cellular processes such as growth, survival, cell proliferation, differentiation, motility, and even cell death [10,11]. It suggested to have a role in breast cancer development and progression [12]. FGFR2 rs2981578 is one of the 14 SNP of FGFR2 located in intron 2 [13].FGFR2 rs2981578 was recognized as a genetic variant linked to breast cancer susceptibility in genome-wide studies (GWAS) [8,9]. It is also noted for its significance in dictating the response to the response to chemotherapy protocols incorporating cyclophosphamide, epirubicin, and docetaxel among breast cancer patients [14]. Evidence suggests that the risk allele contributes to an enhancement in FGFR-2 expression. Hence, genetic variations within the FGFR2 gene have pinpointed it as a potential contributor to the onset of breast cancer.

Multiple studies have explored the association between FGFR-2 rs2981578polymorphism and breast cancer across various patient populations. However, some of these studies did not demonstrate an association. The risk impact of variations discovered within one population may vary in other populations due to diverse underlying factors. Therefore, it is crucial to validate previously associated loci in multiple populations across the world. This would help to identify the genetic diversity of this locus among different populations, particularly in Ethiopia, where the incidence of breast cancer is increasing at an alarming rate. Because of the inconsistent association between this gene polymorphism in different ethnic groups, our study aimed to evaluate and clarify the relationship between breast cancer susceptibility and FGFR2 polymorphism(rs2981578)using a matched case-control study design in Gondar, Northwest Ethiopia.

## Methods

### Study setting, design

Age (±5 years) and sex-matched case-control study between August2022 to April2023 was conducted at the University of Gondar Comprehensive Specialized Hospital (UoGCSH) in Gondar, Northwest Ethiopia, within the Amhara regional state. The University of Gondar Comprehensive Specialized Hospital is one of the largest hospitals in the country. It is a tertiary teaching hospital serving a catchment area of approximately 7.5 million people. It has both pediatric and adult oncology wards. The adult oncology ward serves more than 1500 cancer patients every year [15]. The molecular analysis was conducted at the molecular biology laboratory of the Institute of Biotechnology at the University of Gondar.

### Study participants

All histologically breast cancer patient with age of 20–80 years (as cases) and apparently healthy (as controls) who visit the hospital were considered as the source population. All breast cancer patients who were newly diagnosed at the time of the study period who doesn’t started chemotherapy which visit adult Oncology ward were included as cases, while apparently healthy non breast cancer individuals attending the hospital during the study period were included as controls.

For both cases and controls, individuals with age less than 18 years and greater than 80 years and/or who have clinically confirmed comorbidity such as tuberculosis, diabetes mellitus, liver disease, pregnancy hypertension, renal disease, inflammatory disease, thyroid disease, and secondary breast cancer were excluded.

### Sample size determination and sampling method

Employing G* power software 3.1.9 and opting for an independent t-test, the study’s sample size was computed based on alpha = 0.05, power = 0.8(80%), effect size (d) = 0.5 and allocation ratio N1/N2 = 1, resulting in a sample size of 128. Accounting for a 10% non-response rate, an enrollment targeted 140 participants.

### Data variables

The dependent variable is breast cancer, whereas **FGF Receptor-2rs2981578 SNP**, socio-demographic factors (age, sex, education level, marital status, and residential, etc), behavioral factors (physical exercise, alcohol consumption), anthropometric (BMI, weight to (height)^2^ ratio), and parity was taken as explanatory variables.

### Data collection tool

A structured questionnaire was adapted from the “WHO STEP-wise approach to chronic disease risk factor Surveillance (STEPS)” with little modification (Stepwise-WHO, 2016). The questionnaire was first drafted in English, and subsequently, it underwent translation into the local language (Amharic version). Afterward, language experts conducted a back translation to English to ensure consistency and accuracy in the meaning of words. Two skilled nurses from the oncology unit were selected as data collectors and received training from the principal investigator. Upon completing the data collection process, daily reviews were conducted to confirm the completeness and consistency of each questionnaire and the general data quality. A preliminary test was performed on 5% of the sample population consisting of individuals from another hospital who were not part of the primary study. On-site supervision was conducted by the supervisor and principal investigator.

### Anthropometric measurements

The body weight and height were measured using a portable digital scale and portable stadiometer, respectively. Standing barefoot, the height was measured and reported in meters. The calculation of body mass index (BMI) involved dividing weight (in kilograms) by the square of height (in meters). Participants’ BMI was then used to classify them into different groups; underweight (BMI < 18.5 kg/m^2^), healthy weight (18.5–25 kg/m^2^), overweight (25.0–29.9 kg/m^2^) or obese (≥ 30 kg/m^2^) [16]. Data collectors were oriented for one day on procedures for measuring the weight and height of the study participants and were also made familiar with the questionnaire.

### Blood samples collection and genetic analysis

A 5 ml of blood was collected using EDTA coated tube by certified health care professionals in the Hospital from each participant (cases and controls). Then, the blood samples were kept in a −21ºC refrigerator for genetic analysis through the salting out method.

### Genetic analysis

#### Genomic DNA analysis.

The DNA isolation was done using non-enzymatic salting-out approach [17] by taking EDTA anti-coagulated blood of both patients and healthy controls and transferring it to a sterilized 1.5 ml Eppendorf tube, then red blood cells were removed by lysing them with a buffer solution (TKM 1 containing Tris-KCl-MgCl2, and 1x Triton-X). similarly, white blood cells were lysed using a nuclear lysis buffer solution containing TKM 2 and 10% sodium dodecyl sulfate(SDS) was utilized. Then, a highly concentrated salt solution of 6M NaCl was used to precipitate and eliminate proteins. Subsequently, the DNA was precipitated by freezing with isopropanol and washed with 70% ice-cold ethanol. The genomic DNA was dissolved using Tris-EDTA (TE) buffer and stored at −20 °C till use Verification of both the quality and quantity of the isolated genomic DNA was conducted by examining it on 1.5% agarose gel under UV light, utilizing a gel documentation system (JY04S-3C, UK) as well as by measuring with 2 microliters (2μl) of Ethidium bromide and a Nanodrop reader [18].

#### Polymerase Chain Reaction-Restriction Fragment Length Polymorphism (PCR-RFLP) Technique.

THE (PCR-RFLP) method was employed to determine the FGFR2 rs2981578 genotypes, utilizing specific forward (5’ AATGCTGCTTTGGAGGATTG-3’) and reverse (5’-CCAGAGGACTGAAACCCACA-3’) primers. Within a 25 μl reaction mixture, amplification was undertaken using 12.5 μl of 5.0 X Firepolmaster mix (which includes 12.5mM MgCl2, 1mM dNTPs, 0.4M Tris-HCl, 0.1M (NH4)2SO4, 0.1% w/v Tween-20, and DNA polymerase), together with 1 μl forward primer, 1 μl reverse primer, 2 μl of each sample, and 8.5 μl PCR grade water, ensuring the total volume was achieved. Reactions were carried out in a TC 412 PCR thermocycler (Eppendorf, United Kingdom). The PCR condition was modified according to the master mix condition and primer melting point. At the outset of amplification, there was a 4-minute denaturation phase at 95 °C, succeeded by 35 cycles. In each cycle, denaturation took place for 30 seconds at 95 °C, followed by primer hybridization for 35 seconds at 56.8°C, elongation for 40 seconds at 72 °C, and finally, a 5-minute elongation step at 72 °C concluded the process [19].Two separate PCRs were carried out for every individual DNA sample. The results are summarized in Table 1.

**Table 1:** Primers and a restriction enzyme for genotyping of FGFR2 rs2981578single nucleotide polymorphism.

Locus	Primer sequence	PCR product	RE	SNP	Genotype
rs2981578	F5’AATGCTGCTTTGGAGGATTG-3’	173	SsiI(AciI)	G/A	GG (84,89) AG(173,84,89) AA(173)
	R5’CCAGAGGACTGAAACCCACA-3				

**Note:** F, Forward primer; R, Reverse primer; RE, Restriction Endonuclease.

The PCR products, amplified to a volume of 12 μL, were combined with 3 μL of PCR loading dye (Medax®) and then carefully loaded into wells on an agarose gel. Electrophoresis was subsequently performed in 1X tris acetate EDTA (TAE) buffer, leading to the separation of components through electrophoretic action for a total of 50 minutes at 100V on a 2% Agarose gel. After electrophoresis, 173 band-size fragments were observed by UV transilluminator gel documentation system (JY04S-3C, UK) using Gelpro analyzer version 6.3.To digest the PCR products, a mixture of 10 μL was prepared, comprising 5 μL of PCR product, 1 μL of orange buffer (#B19), 3.5 μL of distilled water, and 1 μL of the SsiI (AciI, #ER1791) restriction enzyme from Thermo Fisher Scientific, U.S. The FGFR2 rs2981578 fragment, amplified to 173 bp, underwent enzymatic digestion at 37°C for 3 hours before being stored at −20°C for 20 minutes. The digestion products underwent electrophoretic separation on a 2.5% agarose gel at 120 volts for 35 minutes. When subjected to digestion, the wild GG genotype breaks into two segments measuring 84 and 89 base pairs. In contrast, the AA genotype, with its mutated form, remains undigested. The AG genotype, representing a heterozygous state, produces three fragments measuring 173, 84, and 89 base pairs [20].Recognition by AciI occurs with the 4-base non-palindromic motif 5’ CCGC 3’, but if a substitution of the “G” nucleotide with an “A” (G/A) happens, the AciI recognition site is lost [21]. To gauge genotyping quality, a portion of 10% of randomly chosen samples was re-genotyped, revealing no disparities.

### Data quality control and assurance

#### Pre-analytical phase.

Blood specimens were aseptically obtained, with adherence to universal safety protocols. The meticulous labeling and packaging of blood-filled EDTA tubes in a suitable container (icebox) were ensured. After ensuring proper packing, all blood samples are transported to the molecular biology laboratory at the Institute of Biotechnology and stored in a −20°C refrigerator. Before beginning sample analysis, maintaining the cleanliness of the laboratory and workbench is ensured. Furthermore, wearing gloves and a lab coat daily is imperative to prevent cross-contamination with human skin.

#### Analytical phase.

Blood samples were extracted within a safety hood following the manufacturer’s guidelines. The PCR-RFLP technique was used to process the samples after optimizing the PCR machine. Reagents and testing methods were validated with known controls, and DNA purity was assessed using a spectrophotometer, targeting a 260/280 nm ratio of 1.8–2.0. An automatic electric balance accurately measured agarose gel powder. The process was overseen by experienced technicians to ensure compliance with standard laboratory protocols.

#### Post-analytical phase.

The reporting underwent double verification before the results were recorded in a registration book, which also included the individual’s daily work charts, to ensure accuracy and eliminate errors in the test outcomes.

### Data processing and statistical analysis

The data obtained from laboratory analyses of the blood samples and questionnaires were checked for completeness and cleaned by coding and entering the data into epi-data-7.2 and then exported to SPSS version 25 for further analysis. Simple descriptive statistics were used to present the socio-demographic and clinical characteristics of the study subjects. The categorical variables were analyzed using the chi-square (χ^2^) test and are expressed as frequency and percentage. Quantitative data was displayed using means and standard deviations (X ± SD), while independent t-test was used to analyze continuous variables between breast cancer patients and controls. To evaluate Hardy–Weinberg equilibrium (HWE), a goodness of fit Chi-square test was employed, where significance at P < 0.05 denoted significant disequilibrium. The study evaluated the relationship between FGFR2 rs2981578 gene polymorphisms and breast cancer risk, employing both co-dominant and per-allele unconditional binary logistic regression models, along with a 95% confidence interval for risk correlations and cumulative risk analysis. Investigation into the Association between FGFR2 rs2981578 and established environmental factors in breast cancer susceptibility involved stratifying cases and controls based on exposure genotype and employing dummy variables for each category. A case-control analysis was utilized to explore how breast cancer patients’ histopathology and clinical traits interact with FGFR2 rs2981578.We examined Association terms in dominant genetic models. Results were deemed statistically significant if the p-value<0.05. Furthermore, graphical representations were utilized to elucidate the findings of RFLP gel electrophoresis.

### Ethical considerations

This research was conducted after the ethical clearance was obtained from the Institutional Review Board (IRB) of University of Gondar, College of Medicine and Health Science, School of Medicine research review, and ethics committee with reference number (Ref. SOM/1801/2022). Prior to beginning data collection, the University of Gondar Comprehensive Specialized Hospital approved the ethical clearance. The IRB waived the consent to do the research and after a suitable explanation of the study’s purpose, advantages, and possible risks, each participant provided verbal informed consent before the actual data collection procedure. Furthermore, confidentiality was guaranteed by omitting their name and mentioning the security of the location where the data would be stored after collection. And the study subject was recruited from June 01-September 01/2022GC.

## Results

### Sociodemographic profiles of study participants

The study included 140 participants, divided equally into two groups: 70 diagnosed with breast cancer and 70 non-cancer controls, matched for age (±5 years) and sex. The age group of 41–50 years constituted the largest segment of participants, making up 32.1% of the total. For cases and controls, it was roughly 50% and 56% of them that were unemployed. For the cases and controls, the mean BMI was 21.93 ± 3.09 kg/m2 and 21.86 ± 3.40 kg/m2, respectively. See Table 2 for Sociodemographic details.

**Table 2:** Sociodemographic profiles of study participants (N = 140) at Gondar Comprehensive Specialized Referral Hospital, Gondar, Northwest Ethiopia.

Variables		Cases (n),%	Control (n),%	Total (N),%	P-value
Age	20-30	8(11.4%)	14(20.0%)	22(15.7%)	0.150
	31-40	19(27.1%)	13(18.6%)	32(22.9%)	
	41-50	19(27.1%)	26(37.1%)	45(32.1%)	
	51-60	17(24.3%)	15(21.4%)	32(22.9%)	
	61-70	7(10.0%)	2(2.9%)	9(6.4%)	
	Mean ± SD	45.09 ± 11.12 years	42.43 ± 10.7 years		0.152
Ethnicity	Amhara	70(100%)	67(95.7%)	137(97.9%)	0.245^A^
	Tigrai	0(0%)	3(4.3%)	3(2.1%)	
Religion	Orthodox	45(64.3%)	40(57.1%)	85(60.7%)	0.231
	Muslim	10(14.3%)	7(10%)	17(12.1%)	
	Other	5(7.4%)	3(4.3%)	8(5.7%)	
Residence	Rural	46(65.7%)	43(61.4%)	89(63.6%)	0.598
	Urban	24(34.3%)	27(38.6%)	51(36.4%)	
Educational level	Primary education	35(50%)	30(42.85%)	65(46.4%)	0.687
	Secondary education	11(15.7%)	16(22.85%	27(19.3%)	
	Higher education	4(5.7%)	3(4.3%)	7(5.0%)	
	Illiterate(none)	20(28.6%)	21(30%)	41(29.3%)	
Occupation	Housewife	25(35.7%)	29(41.4%)	54(38.6%)	0.452
	Government employed	8(11.4%)	12(17.1%)	20(14.3%)	
	Self-employed	5(7.1%)	14(20.0%)	19(13.6%)	
	unemployed	25(35.7%)	27(38.6%)	52(37.1%)	
Marital status	Single	8(11.4%)	10(14.3%)	18(12.9%)	0.581
	Widow	18(25.71%)	15(21.4%)	33(23.6%)	
	Divorced	17(24.3%)	12(17.1%)	29(20.7%)	
	Married	27(38.6%)	33(47.1%)	60(42.9%)	
BMI	<18.5	11(15.7%)	4(5.7%)	9 (6.4%)	0.245
	18.6-25	50(71.4%)	54(77.1%)	91(65%)	
	25-30	7(10%)	8(11.4%)	31(22.1%)	
	>30	2(2.9%)	4(5.7%)	7(5%)	
	Mean ± SD	21.93 ± 3.09 Kg/m square	21.86 ± 3.40 Kg/m square		0.342
Family history	Yes	4(8.7%)	5(7.1%)	9(64.3%)	0.730
	No	66(94.3%)	65(92.9%)	131(5.7%)	

Note: P-value was obtained by Chi-square test;

^A^p-value, Fisher’s Exact Test p-value; *p-value <0.05 is significant. Abbreviation; BMI, Body Mass Index; SD, Standard Deviation.

### Reproductive characteristics of study participants

The majority of cases (70%) experienced menarche after the age of 12 and had three or more children. Among the 63 cases with a history of breastfeeding, 57.1% revealed that their children had been breastfed for more than twenty-four months on average. Of the parous cases, 60.3% had their first child between the ages of 20 and 30.Out of the 41 postmenopausal cases, 70.3% of them had an early onset of menopause, occurring at age forty-five or younger. Between cases and controls, none of the reproductive traits differed significantly (p. value >0.005).See Table 3 for detail Reproductive characteristics.

**Table 3:** Reproductive characteristics of study participants (N = 140) at Gondar Comprehensive Specialized Hospital, Gondar, Northwest Ethiopia.

Variables		Cases (n), (%)	Control (n), (%)	P-value
Age at menarche	≤12	21(30%)	15(24.4%)	0.496
	>12	49(70%)	55(78.6%)	
	Mean *± SD*	13.54* ± *1.501	13.81* ± *1.516	0.289
Parity	0(nulliparous)	7(10%)	5(7.1%)	0.553
	1-2	14(20%)	19(27.1%)	
	>2	49(70%)	16(65.7%)	
	Mean *± SD*	4.06* ± *2.30	4.16* ± *1.67	0.768
Contraception history	Yes	59(84.3%)	47(67.1%)	0.018
	No	11(15.7%)	23(32.9%)	
Breastfeeding	Yes	63(90%)	65(92.9%)	0.546
	No	7(10%)	5(7.1%)	
Breastfeeding duration ^a^	≤ 24 months	21(33.3%)	27(41.5%)	0.526
	>24 months	42(66.7%)	38(58.5%)	
	Mean *± SD*	23.81* ± *3.02	24.23* ± *3.64	0.478
Age at birth of first child^birth^	10-20	20(31.7%)	26(40%)	0.669
	21-30	38(60.3%)	33(50.8%)	
	31-40	5(7.9%)	6(9.2%)	
	Mean *± SD*	20.30 *± *5.031	20.35* ± *5.800	0.957
Menopausal status	Premenopausal	29(41.4%)	33(47.1%)	0.496
	Postmenopausal	41(58.6%)?	37(52.9%)?	
Age at menopause	≤ 45	29(70.3%)	24(64.9%)	0.384
	>45	12(29.3%)	13(35.13%)	
	Mean *± SD*	43.78* ± *3.785	43.86* ± *4.008	0.924

Note: P-value was obtained by the chi-square test; *p-value <0.05 is considered statistically significant. Breast feeding duration is a percentage calculated from the total number of women who have a breastfeeding history. Age at birth of first child^b^irth, the percentage calculated from a total parous woman. Age at menopause, the percentage was calculated from the total of postmenopausal women. Abbreviation: SD, Standard Deviation.

### Histopathological and clinical characteristics of breast cancer patients

The study found that the majority of patients examined displayed ductal carcinoma (75.7%), while 34.3% of them were identified as having stage-IV breast cancer. Among the 67 individuals diagnosed with invasive cancer, 43.3% were found to have a grade III tumor according to the Nottingham grading system **See Table 4 for detail Tumor Characteristics.**

**Table 4:** Tumor characteristics in breast cancer patient (n = 70) at Gondar Comprehensive Specialized Hospital, Gondar, Northwest Ethiopia.

Variables		Frequency(N), %
Age at diagnosis	Mean *± SD*	45.09 ± 11.12 years
Histological type	Ductal carcinoma	53(75.7%)
	Ductal carcinoma in situ	3(4.3%)
	Lobular carcinoma	4(5.7%)
	Breast cancer no special type (BCNST)	8(11.4%)
	Mucinous carcinoma	1(1.4%)
	papillary carcinoma	1(1.4%)
TNM stage	Stage 0	3(4.3%)
	Stage I	2(2.9%)
	Stage II	14(20.0%)
	Stage III	27(38.6%)
	Stage IV	24(34.3%)
Tumour size	Tis	3(4.3%)
	TI	2(2.9%)
	TII	13(18.6%)
	TIII	27(38.6%)
	TIV	25(35.7%)
Lymph node status	N0	18(25.7%)
	N1	12(17.1%)
	N2	14(20.0%)
	N3	26(37.1%)
Metastasis	M0	46(65.7%)
	M1	24(34.3%)
Histological Grade^s^	GI	10(14.9%)
	GII	29(43.3%)
	GI	28(41.8%)
Chief complaint	Breast pain	3(4.3%)
	Ulceration	15(21.4%)
	Palpable breast mass	52(74.3%)
Lateralization	Right breast	45(64.3%)
	Left breast	21(30%)
	Bilateral	4(5.7%)
Duration of symptoms	<4 months	1(1.4%)
	5-8	18(25.7%)
	9-16	26(37.2%)
	17-24	18(25.7%)
	≥25	7(10%)

Note: ^b^ histologic grade is done only for invasive breast cancer (N = 67).^a^TNM-based cancer staging system: AJCC 8th Ed. TNM: T, Tumor size; N, lymph node; M, Metastasis, Grade; SD, Standard Deviation.

### FGFR2 rs2981578 genotypes of study participants

**Fig 1:**
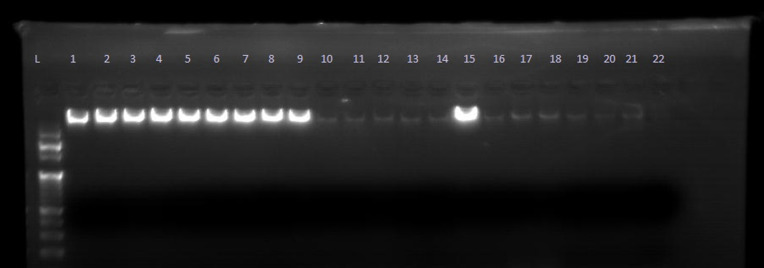
Electrophoretic pattern for DNA presence [1.5% agarose gel (L = 100 BP standard DNA ladder, Lane 1-9 DNA sample, Lane 10-13 NC = Negative Control (all mixture without DNA sample)].

**Fig 2:**
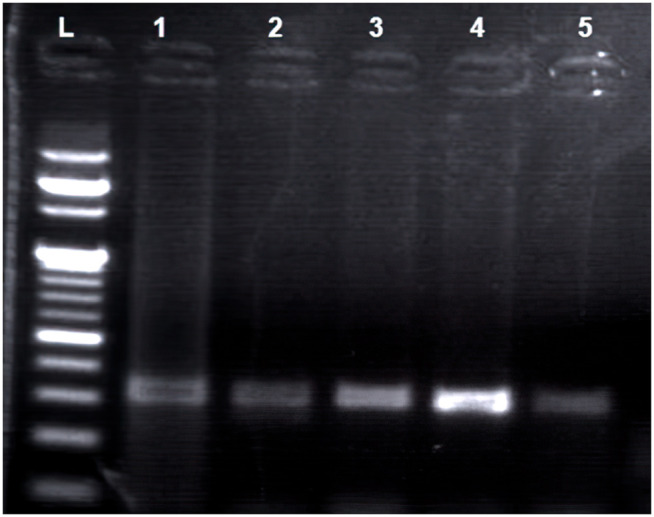
Electrophoretic pattern of PCR products of FGFR2 rs2981578 (2% gel, L = 1- 50 BP standard DNA ladder; Lane 1-4 = 173 BP).

Conventional PCR-RFLP techniques were employed to analyze DNA extracted from blood samples of 140 individuals, comprising 70 cases and 70 controls. DNA presence was verified through electrophoresis on a 1.5% agarose gel stained with Ethidium bromideas shown in Fig 1. The pattern of amplification of PCR products of FGFR2 (rs2981578) with 173 base pairs was shown in a 2% agarose gel as shown in Fig 2. Restriction endonuclease (SsiI/AciI)-digested and undigested products were shown in a 2.5% agarose gel.

**Fig 3:**
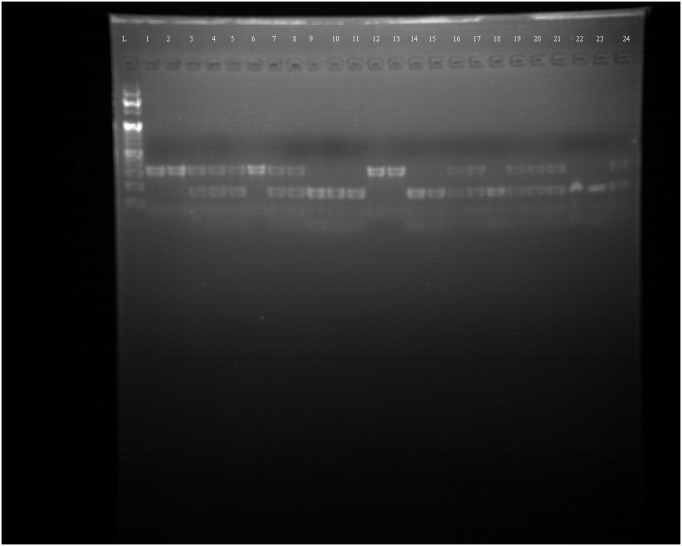
Representative Agarose gel image for restriction analysis of Rs2981578 [2.5% gel, digestion with *AciI* enzyme: L = 1- 50 BP standard DNA ladder; Lane 1 = 173 BP (AA); Lane 2 = 173, 89, 84 BP (AG) and Lane 3 = 89, 84 BP (GG); Lane 1-5 are cases and Lane 6-10 are controls).

Electrophoretic pattern of PCR products of FGFR2 rs2981578 (2% gel, L = 1–50 BP standard DNA ladder; Lane 1–4 = 173 BPSuccessfully amplified samples with the conventional PCR were processed in restriction digestion with *AciI* restriction endonuclease for SNP analysis in agarose gel electrophoresis. The interpretation of the RFLP digestion outcomes was as follows: Samples exhibiting a single band (173 bp) were categorized as mutant (Homozygous recessive, AA); those showing one band consisting of 84 bp and 89 bp fragments were identified as wild (Homozygous Dominant, GG) type; while samples displaying two bands representing 173 bp, 84 bp, and 89 bp digested products were classified as heterozygous (AG) **as shown in Fig 3**.

Genotype frequencies for FGFR2 rs2981578 in cases and controls conformed to Hardy-Weinberg equilibrium (p = 0.399, p = 0.993) as shown in Table 5.

**Table 5:** Hardy-Weinberg equilibrium at Gondar Oncology Center for cases and controls.

Gene	SNP ID	Cases(n = 70)			Controls (n = 70)		
		MA	MAF	HWE ^*^p-value	MA	MAF	HWE ^*^p-value
FGFR2	Rs2981578	A	58%	0.399	A	42%	0.993

Note: The p-value was calculated using a chi-square test for goodness of fit;

* p-value >0.05 indicates HWE equilibrium. Abbreviations: MA (Minor Allele), MAF (Minor Allele Frequency), HWE (Hardy-Weinberg Equilibrium).

A significant variation (p < 0.05) was observed in the distribution of the FGFR2 rs2981578 genotype polymorphism between the two groups The distribution of FGFR2 rs2981578 genotypes among cases and controls revealed notable differences. Among the cases, 21.4% carried the “GG” genotype, 41.4% had the “AG” genotype, and 37.1% exhibited the “AA” genotype. In contrast, the control group showed a genotype distribution of 32.9% for “GG”, 50.0% for “AG”, and 17.1% for “AA”. Using the “GG” wild type genotype as the reference, the “AG” genotype was associated with a non-significant increase in breast cancer risk (COR = 1.27; 95% CI: 0.562–2.872; p = 0.565). However, individuals with the “AA” genotype demonstrated a statistically significant association with increased breast cancer risk (COR = 3.30; 95% CI: 1.293–8.538; p = 0.013). When the variant genotypes AG and AA were combined (AG + AA), they were found in 78.6% of cases and 67.1% of controls, with a non-significant association with an increased risk of breast cancer (COR = 1.79; 95% CI: 0.841–3.829, p = 0.131). At the allele level, the “A” allele was significantly more frequent among cases (58%) compared to controls (42%), and was associated with an increased risk of breast cancer (COR = 1.88; 95% CI: 1.173–3.029; p = 0.004), whereas the “G” allele was more prevalent in the control group (58%) and was used as the reference. See Table 6 for detail Genotype Characteristics of cases and controls.

**Table 6:** Genotype comparison: breast cancer patients versus controls at Gondar Comprehensive Specialized Hospital, Northwest Ethiopia.

Genotype& allele	Cases (n), (%)	Control (n), (%)	P-value	COR(CI)(95%CI)	P-value
**FGFR2 rs2981578**					
**Genotype**					
GG	15(21.4%)	23(32.9%)		Ref	
AG	29(41.4%)	35(50%)		1.27(0.562-2.872)	0.565
AA	26(37.1%)	12(17.1%)	0.025^*^	3.30(1.293-8.538)	0.013^*^
AG + AA	55(78.6%)	47(67.1%)	0.128	1.79(0.841-3.829)	0.131
**Allele**					
G	59(42%)	81(58%)	0.009	Ref	
A	81(58%)	59(42%)		1.88(1.173,3.029)	0.004^*^

Note: The genotypes were stratified according to Multiplicative (homozygous recessive, heterozygous recessive versus homozygous dominant) and Dominant genetic analysis model (homozygous recessive and heterozygous recessive versus homozygous dominant). A binary logistic regression test was used to determine the association for case versus control analysis;

* p-value below 0.05 was regarded as significant-values indicate the chi-square p-value. P-values indicate binary logistics odds ratio p-value. **Abbreviations; Ref** denotes the reference point, **CI** stands for confidence interval, and **COR** represents the crude odds ratio

### Association FGFR2 rs2981578 and clinicopathological characteristics of breast cancer patients

The association between various FGFR2 rs2981578 genotypes and the stratified nature of tumors demonstrated a statistically insignificant (p > 0.05) variation in the occurrence of GG and AG + AA within both histopathological and clinical characteristics of breast cancer (COR = 3.41, 95% CI: 1.07–10.88, p = 0.038) as shown in Table 7.

**Table 7:** FGFR2rs2981578 SNP and breast cancer characteristics at Gondar Oncology Center, Northwest Ethiopia.

Variables		*FGFR2* rs2981578 Genotype (N)		P-value	COR(95%CI)
		GG(15)	AG + AA(55)		
Age at diagnosis	>45	9	27		Ref
	≤45	6	28	0.456	1.56(0.487-4.964)
TNM stage	0- II	5	14		Ref
	III – IV	10	41	0.038^*^	3.41(1.07-10.88)
Tumour size	Tis-TII	5	13		Ref
	TIII-TIV	10	42	0.449	1.62(0.467-5.586)
Lymph node	N0-N1	3	27		Ref
	N2-N3	12	28	0.054	0.26(0.066–1.021)
Metastasis	M0	9	37		Ref
	M1	6	18	0.525	0.73(0.225–2.367)
Grade^α^	GI-GII	6	36		Ref
	GIII	9	19	0.081	0.352(0.1089-1.137)

Note: The genotypes were stratified according to the dominant genetic analysis model which compares genotypes having at least one mutant allele with wild type genotype as a reference. A binary logistic regression test was used to determine genotype-phenotype association for case-only analysis;

* p-value <0.05 was considered significant. Abbreviations; G, Grade; N, Node; T, Tumor size, Metastasis, Grade^α^: Grade only done for invasive breast cancer.

### The association of FGFR2 rs2981578 G/A genotypes with sociodemographic variables

The relationship between FGFR2 rs2981578 G/A genotypes and sociodemographic variables did not yield significant variations among cases and controls, suggesting that the Association of genotypes with sociodemographic traits was not significant in either group. Women with at least one risk allele A had no difference in the distribution of sociodemographic characteristics between the case and controls (p > 0.05) **as shown in Table 8**.

**Table 8:** FGFR2 rs2981578 G/A genotype association in breast cancer patients vs. controls.

		*FGFR2* rs2981578 Genotype (N)					
		GG(38)			AG + AA(102)		
Variables		case/ controls	P-value	COR(95%CI)	case/ controls	P-value	COR(95%CI)
Age	<49	5/5		Ref	40/45	0.430	0.89(0.240-3.297)
	≥ 49	18/10	0.215	1.80(0.418-7.757	18/10	0.215	1.80(0.418-7.757)
Residence	Rural	9/5		Ref	37/38	0.154	0.54(0.166-1.766)
	Urban	6/18	0.021^*^	0.19(0.04-0.76)	18/9	0.125	2.05(0.819-5.151)
Educational level	Literate	14/22		Ref	36/27	0.041	2.09(0.909-4.831)
	Illiterate	1/1	0.756	1.57(0.09-27.21)	19/20	0.408	0.71(0.320-1.589)
Employment	Employed	3/10		Ref	10/16	0.171	2.08(0.459-9.458)
	Unemployed	12/13	0.145	3.07(0.68-13.92)	45/31	0.0704	2.32(0.932-5.787)
Marital status	Married	5/7		Ref	22/26	0.397	1.18(0.329-4.262)
	Other	10/16	0.851	0.88(0.22-3.53)	33/21	0.1239	1.86(0.84–4.09)
BMI	≤ 25 Kg/m^2^	13/16		Ref	48/42	0.795	1.40(0.607-3.261)
	>25 Kg/m^2^	3/6	0.544	0.62(0.13-2.95)	6/6	0.828	0.88(0.26–2.92)

Note: The genotypes were stratified according to the dominant genetic analysis model which compares genotypes having at least one mutant allele (AG + AA) with wild type(GG)genotype as a reference. A binary logistic regression test was used to determine genotype-phenotype association for case versus control analysis;

* the significance was set at p-value< 0.05, indicating statistical significance. Abbreviations; BMI, Body Mass Index

### Association of FGFR2 rs2981578 G/A genotypes with behavioral characteristics

Women who have at least one risk allele A did not display any variation in the distribution of behavioral characteristics between cases and controls, as indicated by a p-value>0.05See Table 9 for more details.

**Table 9:** The FGFR2 rs2981578 G/A genotypes and behavioral characteristics in breast cancer patients and non-cancer controls.

		*FGFR2* rs2981578 Genotype (N)					
		GG(38)			AG + AA(102)		
Variables		case/ controls	P-value	COR(95%CI)	Case/ controls	P-value	COR(95%CI)
Physical exercise	Yes	8/6		Ref	8/12	0.980	0.50(0.125-1.999)
	No	8/16	0.157	0.38(0.097-1.456)	46/36	0.258	1.92(0.708-5.185)
Alcohol consumption	No	5/4		Ref	14/9	0.392	1.24(0.262-5.915)
	Yes	16/13	0.984	0.98(0.219-4.434)	35/44	0.166	0.51(0.198-1.319)

Note: The genotypes were stratified according to the dominant genetic analysis model which compares genotypes having at least one mutant allele with wild-type genotype as a reference. A binary logistic regression test was used to determine genotype-phenotype association for case and control analysis; *A significant p-value was < 0.05

### Association of FGFR2 rs2981578 G/A genotypes with reproductive characteristics

The study assessed how variations in the FGFR2 rs2981578 gene influence the likelihood of developing breast cancer among women with varying reproductive traits. The findings revealed that women carrying at least one risk allele A showed no difference in the distribution of reproductive traits between cases and controls (p-value>0.05) as shown in Table 10.

**Table 10:** The FGFR2 rs2981578 G/A genotypes and reproductive characteristics in breast cancer patients and non-cancer controls.

		*FGFR2* rs2981578 Genotype (N)					
		GG(38)			AG + AA(102)		
Variables		case/ controls	P- value	COR(95%CI)	case/ controls	P-value	COR(95%CI)
Parity	parous	15/20		Ref	48/45	0.189	1.42(0.650-3.113)
	nulliparous	2/1	0.441	2.67(0.22-32.23)	5/4	0.821	1.17(0.296-4.641)
Age at menarche	>12	13/18		Ref	36/37	0.245	1.34(0.577-3.146)
	≤12	2/5	0.517	0.55(0.09-3.31)	19/10	0.142	1.95(0.800-4.68)
First childbirth age	≤ 25 >25	14/19 2/3	0.919	Ref 0.91(0.13-6.16)	46/42 7/7	0.167 0.874	1.48(0.663-3.332) 0.91(0.296-2.820)
Breastfeeding	Yes	13/20		Ref	50/45	0.096	1.70(0.763-3.828)
	No	3/2	0.393	2.31(0.33-15.75)	4/3	0.818	1.20(0.255–5.655)
Contraception history	No	8/8		Ref	3/15	0.060	0.20(0.041-1.971)
	Yes	11/11	0.99	1.0(0.276-3.625)	48/36	0.059	6.67(0.79–24.78)
Menopausal status	postmenopause	9/4		Ref	32/33	0.097	0.43(0.121-1.541)
	premenopause	9/16	0.058	0.25(0.05-1.04)	20/17	0.640	1.21 (0.540-2.725)
Age at menopause	≤ 45	8/9		Ref	21/15	0.221	1.57(0.494-5.025)
	>45	10/11	0.973	1.02(0.28-3.681)	2/2	0.750	0.71(0.090-5.655)

Note: The genotypes were stratified according to the dominant genetic analysis model which compares genotypes having at least one mutant allele (AG + AA) with wild-type genotypes (GG and CC) as a reference. A binary logistic regression test was used to determine genotype-phenotype association for case and control analysis; *p-value <0.05 was considered significant.

## Discussion

Breast cancer, being the highest-ranking among different types of cancers, has a catastrophic impact on women worldwide. Several polymorphic loci were recently identified by two independent GWAS as being associated with breast cancer risk. Among many more, one of the strongest associations was found in the FGFR2gene. This study explores how FGFR2 rs2981578 SNP and various risk factors intersect in breast cancer among Ethiopians in Gondar, Amhara region. Many scholars have asserted that the FGFR2 gene is linked to an increased likelihood of breast cancer in American, African American, European, and Asian populations. However, there is a notable absence of research investigating these potential correlations within the Ethiopian demographic. Seventy breast cancer patients and an equal number of controls, totaling 140 study participants, were included in the study. The groups exhibited comparable age distributions and means, indicating successful matching. It is noteworthy that there were no remarkable differences detected in sociodemographic, behavioral, or reproductive traits between the cases and controls.

The distribution of genotypes for the rs2981578 polymorphism among cases and controls adhered to the Hardy-Weinberg equilibrium, as indicated by p-values of 0.399 and 0.993, respectively. Our study revealed a significant elevation in the occurrence of the “AA” genotype and “A” allele among breast cancer patients in comparison to healthy controls. The AA genotype was observed in 37.1% of breast cancer cases compared to 17.1% of controls, while the A allele was present in 58% of cases and 42% of controls. Statistical analysis demonstrated substantial differences, with chi-square values of 0.025 for “AA” genotype and 0.009 for “A” allele, respectively. When rs2981578 was examined as a categorical variable using the wild genotype (GG) as the reference category, individuals with the AA genotype displayed a risk of developing breast cancer more than three times higher than those with the GG genotype (OR 3.30; 95% CI 1.293–8.538; p = 0.013). Additionally, individuals carrying the A allele were at a higher risk of breast cancer compared to those with the G allele (OR: 1.88; 95% CI: 1.173–3.029; p = 0.004).

The findings are in harmony with a meticulous genotype mapping study involving 1253 African American invasive breast cancer individuals and 1245 controls. This investigation demonstrated a notable correlation between the prevalence of the minor allele A and the risk of breast cancer for rs2981578 (per-allele COR, 1.20: 95% CI, 1.03–1.41: p_trend_ = 0.02) [22], demonstrating odds ratio estimates similar to those seen European [8]and Asian population [23], akin to the findings of GWAS research. Similarly, a meta-analysis encompassing three Asian studies, comprising 833 cases and 1012 controls, also demonstrated a notable correlation across various genetic models. For instance, in the Allele model, the odds ratio (OR) stood at 1.29 with a 95% confidence interval (CI) of 1.13–1.47, and a p-value of 0.0002. Moreover, under the Dominant model, the OR was 1.71, with a 95% CI of 1.32–2.21, and a p-value of less than 0.0001 [24]. In a study conducted in North India, an association was found between rs2981578SNP and breast cancer susceptibility. The odds ratios (OR) were reported as 1.661 (95% CI: 1.108–2.489) and 1.613 (95% CI: 1.124–2.314) for co-dominant and dominant models, respectively, with corresponding p-values of 0.014 and 0.009 [18].

One potential explanation for the link between the FGFR2 rs2981578 AA genotype and breast cancer, both in our investigation and in studies conducted on different populations, might be attributed to differences in FGFR2 expression. Several studies have done the mRNA or protein expression of FGFR2 in breast cancer. The expression level varies and is influenced by genetic, epigenetic, and molecular factors. Elevated mRNA or protein expression of FGFR2 depends was seen in SUM-52PE cell line [25] and associates with poor prognosis [26]. On the other hand, some of them reported lower FGFR2 expression levels in tumor tissues compared to adjacent normal breast ducts. This could be due to loss of heterozygosity or methylation affecting the FGFR2 locus [27]. These discrepancies highlight the complexity of FGFR2’s role in breast cancer.

Studies focusing on biochemical and structural aspects indicate a marked increase in FGFR2 messenger RNA (mRNA) expression within breast cancer tissues compared to their normal counter parts. The AA genotype in estrogen receptor-positive tumors led to changes in the DNA binding capability of transcription factors such as Oct-1/Runx2 and C/EBPb [28–30]. The binding of heterozygous alleles to FOXA1 and ERα is mirrored by allele-specific changes in chromatin accessibility and the recruitment of RNA polymerase II to a region believed to be an enhancer [31,32].However, even with the proposed potential pathways connecting FGFR2 rs2981578 to the risk of breast cancer, the precise mechanism underlying the development of breast cancer associated with this genetic variant remains unclear.

Conversely, a broader study involving 7,800 postmenopausal African American women, of whom 316 were diagnosed with invasive breast cancer, found that rs2981579, a SNP closely related to rs2981578, displayed a statistically insignificant negative correlation (HR for the G allele 0.99, 95% CI: 0.83–1.17, p = 0.87) among women aged 50–79 [33]. Moreover, findings from a population-based case-control study, which included 5,761 unrelated African-American women from 11 epidemiological studies, also showed consistent results [34]. Among 2,594 African-American women examined through a meta-analysis, which encompassed 810 cases and 1,784 controls, a mutation proximal to rs2981578 (G > A), known as rs2981578 (C > T), exhibited a potential decrease in breast cancer risk, particularly among those with the TT genotype. The odds ratio (OR) for TT versus CC/CT was 0.55 with a 95% confidence interval (CI) of 0.38–0.79, 0.51 (95% CI: 0.35–0.76) for TT versus CC, and 0.58 (95% CI: 0.40–0.85) for TT versus CT. This trend was notably more prominent in the Asian population [35].Compared to the results of the present study, other studies conducted in populations from Taiwan [20], Japan [36], and China [37] failed to uncover any notable correlation between the G/A polymorphism of the FGFR2rs2981578 gene and the risk of breast cancer. Inconsistent findings concerning the influence of FGFR2 gene G/A polymorphisms on breast cancer risk may be attributed to the heterogeneity of populations studied, variations in ethnic backgrounds and geographic locations, and potential sampling biases. Various lifestyle factors, including diet and exercise, are also linked to changes in the epigenetic state. Hence, the disparities noted in correlation analyses regarding the FGFR2 G/A polymorphism and breast cancer across various ethnic groups could arise from the interconnections between epigenetic modifications and polymorphism, thereby elucidating the intricate nature of genetic composition [38].

The study of the pathological characteristics of *FGFR2* rs2981578 genotype groups in breast cancer patients has lifted the veil on potential links between *FGFR2* rs2981578 variants and tumor histopathological features. Our research delved into the pathological attributes observed in cancer patients with GG and AG + AA genotypes. The objective was to ascertain whether genetic risk factors for breast cancer correlate with distinct histopathological subtypes, encompassing TNM staging, tumor dimensions, nodal involvement, metastasis, grade of malignancy, and age at diagnosis. Our findings highlight a robust link between the A allele and the development of more aggressive tumor types. Individuals carrying the A allele (AG + AA) are at a notable threefold increased risk of late-stage breast cancer (COR = 3.41, 95% CI: 1.07–10.88, p = 0.038) compared to those with the ancestral genotype (GG).

Hinting at the possibility that the FGFR2 rs2981578 polymorphism may heighten FGFR2’s transcriptional activity or expression as breast cancer advances, underscoring the potential prognostic value of this genetic variation in breast cancer prognosis [28,39]. This contradicts findings that presented more compelling evidence for increasing susceptibility to low-grade subtypes [18,40].Discrepancies in the prevalence of allele A could explain the inconsistency in the Association term observed in both studies.

The present study also examines the Association of FGFR2 rs2981578G/A Genotypes with socio-demographic, behavioral, and reproductive risk factors. Stratified analysis showed a statistically non-significant Association between those factors and rs2981578 polymorphism. Among individuals carrying at least one risk allele (AA + AG genotype) and exhibiting diverse risk factors, the contrast between cases and controls did not reach statistical significance (p > 0.05).The study found no notable divergence in how each locus impacted the risk of breast cancer between pre and postmenopausal women in Japan, as evidenced by the analysis of 697 female breast cancer patients and matched 1,394 controls [36]. Another study in India also reported a non-significant Association of rs2981578 and menopausal status with OR =1.848; 95%CI, (0.846–4.035); p = 0.123 [18]. The exact mechanisms by which FGFR2 rs2981578 polymorphism jointly with demographic and lifestyle factors influence breast cancer development are complex and not yet fully understood. However, several studies have proposed possible mechanisms that may explain the relationship [41]. Therefore, additional investigation is warranted to authenticate and substantiate this finding.

### Strengths and limitations

Through the case-control study design employed, the current research facilitates the exploration of multiple risk factors within distinct populations. Our study selected cases at random and controls from the general population, thereby strengthening the reliability and applicability of our findings. A significant majority (95%) of participants responded promptly to both questionnaire completion and blood sample provision. Additionally, matching was utilized to counteract potential confounding factors related to age and gender. The study was confined to a particular area, encompassing a small geographical region with a consistent genetic composition. Consequently, it is advisable to conduct the task over a broad scope with a larger sample size. Additionally, no assessment was made of the plasma levels of FGFR2 expression and mRNA level, which directly correspond to the genetic variation under examination in this research. Considering the inherent constraints of retrospective case-control studies, our findings should be approached cautiously, especially those beyond the genetic data. We recommend future studies to apply higher-resolution and more advanced SNP genotyping methods for improved accuracy. Furthermore, the extent of our study sample was restricted, particularly when analyzed according to different risk factors. Our conclusions need to be confirmed through validation in a larger sample size. To better grasp the contribution of the FGFR2rs2981578 variant to breast cancer pathogenesis, it’s imperative to examine its correlation with other SNPs both within the same gene and across different genes. Moreover, it will boost the accuracy of predicting risks. Large, meticulously planned studies are essential, incorporating a variety of ethnicities and factors that contribute to risk. This aims to confirm the relevance of these gene SNPs in breast cancer and to evaluate their potential utility as biomarkers for diagnosing the disease, predicting outcomes, or identifying therapeutic targets.

## Conclusion

The current association study in the Ethiopian population demonstrated no significant differences between breast cancer cases and non-cancer control groups about socio-demographic, behavioral, and reproductive characteristics. It also showed a distinct genetic predisposition in comparison to those reported from other populations. High-risk breast cancer susceptibility was connected to the AA genotype and A allele of the FGFR2 rs2981578 G/A gene. Despite stratification by genotypes, the distribution differences between cases and controls did not achieve statistical significance. This could imply a greater burden of disease and genotype-phenotype discrepancies due to disparities in genetic background, including differences in allele frequencies and gene–environment Association.

## Supporting information

S1 File Gel images. Raw electrophoresis gel images supporting including DNA presence patterns [S1-fig1], FGFR2 rs2981578 genotyping PCR products (2% gel)[S2-fig2] and Rs2981578 restriction analysis (2.5% gel)[S3-fig3].
(RAR)

## Abbreviation:

AA: Adenine
BC: Breast Cancer
BMI: Body Mass Index
bp: Base Pair
BRCA: Breast Cancer gene
DNA: Dinucleotide Acid
EDTA: Ethylene Diamine Tetra Acetic Acid
ER: Estrogen Receptor
FGFR: Fibroblast Growth Factor Receptor
FTP: Full-Term Pregnancy
GWAS: Genome Wide Association Studies
PCR: Polymerase Chain Reaction
RFLP: Restriction Fragment Length Polymorphism
SNP: Single Nucleotide Polymorphism
TKM: Tris-KCl-MgCl2
UoGCSH: University of Gondar comprehensive Specialized Hospital.
